# Building capacity to use legal tools to address climate change through a public health lens

**DOI:** 10.1093/eurpub/ckac131.150

**Published:** 2022-10-25

**Authors:** D Patterson, A Garde

**Affiliations:** 1 Groningen Centre for Health Law, University of Groningen, Groningen, Netherlands; 2 Law and Non-comminicable Diseases Unit, University of Liverpool, Liverpool, UK

## Abstract

**Background:**

EPH Conferences have an increased focus on the health impacts of climate change. However, until 2021 EPHC abstracts did not address opportunities to challenge government inaction in national and international courts. ASPHER’s European List of Core Competences for the Public Health Professional (2018) only mentions NCDs and data protection law. Yet litigation to address environmental degradation and climate change is well-developed in Europe and beyond. International environmental law was presciently framed as part of global health law by Joaquin Cayon in Laaser U. and Beluli F. A Global Public Health Curriculum. South Eastern European Journal of Public Health (2016).

**Objectives:**

1. Clarify the role of litigation in achieving environmental and climate goals

2. Highlight the role of public health practitioners (PHP) in documenting evidence of harms

3. Propose ways to build PHP capacity to use litigation to address related public health challenges

**Results:**

In 2021 EUPHA-LAW, EUPHA-ENV, the Faculty of Public Health (FPH) (UK) and partners co-hosted a multi-stakeholder, interdisciplinary, open-access webinar on public health, climate change and litigation with experts from WHO, Greenpeace, ClientEarth, Lancet Countdown and the Groningen Centre for Health Law (GCHL). A related workshop was held at the 14th EPHC. In March 2022, EUPHA-LAW, FPH, GCHL and the University of Liverpool hosted a webinar on climate action in the European Court of Human Rights. [Patterson et al, Post COP26: legal action now part of public health’s environment and climate change toolbox, EJPH 31/5/2022.] EUPHA-LAW is preparing a toolkit for public health practitioners and a conference on public health litigation in 2023.

**Conclusions:**

There is keen interest in the public health community to better understand how litigation can address public health challenges including climate change and environmental degradation. Capacity building is needed.

**Key messages:**

• Climate litigation supported by interdisciplinary collaboration can strengthen government resolve and accountability for action to address environmental and climate commitments.

• Litigation can advance public health objectives even without a favourable court decision. Its value also includes the publicity and awareness generated and engagement of multiple stakeholders.

